# Health Tract, No. 94

**Published:** 1865

**Authors:** 


### HEALTH TRACT, No. 94.

### HOUSEKEEPING HINTS.

HEALTH is impaired, and even life lost sometimes, by using imperfect, unripe, musty, or decaying articles of food. The same money's worth of a smaller amount of good is more nutritious, more healthful, and more invigorating than a much larger amount of what is of an inferior quality. Therefore, get good food, and keep it good until used. Remember that

Fresh meats should be kept in a cool place, but not freezing or in actual contact with ice.

Flour and meal should be kept in a cool, dry place, with a space of an inch or more between the floor and the bottom of the barrel.

SUGARS.—Havana sugar is seldom clean, hence not so good as that from Brazil, Porto Rico, and Santa Cruz. Loaf, crushed, and granulated sugars have most sweetness, and go further than brown.

Butter for winter use should be made in mid-autumn.

Lard that is hard and white, and from hogs under a year old, is best.

Cheese soft between fingers is richest and best. Keep it tied in a bag hung in a cool, dry place. Wipe off the mold with a dry cloth.

Rice, large, clean, and fresh-looking is best.

Sago, small and white, called "Pearl," is best.

Coffee and tea should be kept in close canisters, and by themselves. Purchase the former green; roast and grind for each day's use.

Apples, oranges, and lemons keep longest wrapped close in paper, and kept in a cool, dry place. Thaw frozen apples in cold water.

Bread and cake should be kept in a dry, cool place, in a wooden box, aired in the sun every day or two.

All strong-odored food should be kept by itself, where it can not scent the house.

Bar-soap should be piled up with spaces between them in a dry cellar, having the air all around it to dry it for months before using; the drier, the less waste.

Cranberries kept covered with water will keep for months in a cellar.

Potatoes spread over a dry floor will not sprout. If they do, cut off the sprouts often. If frozen, thaw them in hot water, and cook at once. By peeling off the skin after they are cooked, the most nutritious and healthful part is saved.

Corned beef should be put in boiling water, and boil steadily for several hours.

Hominy or "samp" should steep in warm water all night, and boil all next day in an earthen jar surrounded with water.

Spices and peppers should be ground fine, and kept in tin cans in a dry place A good nutmeg "bleeds" at the puncture of a pin. Cayenne pepper is better for all purposes of health than black.

Beans, white, are the cheapest and most nutritious of all articles of food in this country. The best mealy potatoes sink in strong salt water.

Hot drinks are best at meals; the less of any fluid the better. Any thing cold arrests digestion on the instant.

It is hurtful and is a wicked waste of food to eat without an appetite.

All meats should be cut up as fine as a pea, most especially for children. The same amount of stomach-power expended on such a small amount of food, as to be digested perfectly without its being felt to be a labor, namely, without any appreciable discomfort in any part of the body, gives more nutriment, strength, and vigor to the system, than upon a larger amount, which is felt to require an effort, giving nausea, fullness, acidity, wind, etc.

Milk, however fresh, pure, and rich, if drunk largely at each meal, say a glass or two, is generally hurtful to invalids and sedentary persons, as it tends to cause fever, constipation, or biliousness.

